# Plasma amino acid levels are elevated in young, healthy low birth weight men exposed to short‐term high‐fat overfeeding

**DOI:** 10.14814/phy2.13044

**Published:** 2016-12-12

**Authors:** Amalie Ribel‐Madsen, Lars I. Hellgren, Charlotte Brøns, Rasmus Ribel‐Madsen, Christopher B. Newgard, Allan A. Vaag

**Affiliations:** ^1^ Department of Biotechnology and BiomedicineTechnical University of DenmarkKongens LyngbyDenmark; ^2^ Department of Endocrinology, Diabetes and MetabolismRigshospitaletCopenhagen University HospitalCopenhagenDenmark; ^3^ Danish Diabetes AcademyOdenseDenmark; ^4^ Sarah W. Stedman Nutrition and Metabolism Center and Duke Molecular Physiology InstituteDuke UniversityDurhamNCUSA

**Keywords:** Amino acids, high‐fat overfeeding, insulin resistance, low birth weight, type 2 diabetes

## Abstract

Low birth weight (LBW) individuals exhibit a disproportionately increased, incomplete fatty acid oxidation and a decreased glucose oxidation, compared with normal birth weight (NBW) individuals, and furthermore have an increased risk of developing insulin resistance and type 2 diabetes. We hypothesized that changes in amino acid metabolism may occur parallel to alterations in fatty acid and glucose oxidation, and could contribute to insulin resistance. Therefore, we measured fasting plasma levels of 15 individual or pools of amino acids in 18 LBW and 25 NBW men after an isocaloric control diet and after a 5‐day high‐fat, high‐calorie diet. We demonstrated that LBW and NBW men increased plasma alanine levels and decreased valine and leucine/isoleucine levels in response to overfeeding. Also, LBW men had higher alanine, proline, methionine, citrulline, and total amino acid levels after overfeeding compared with NBW men. Alanine and total amino acid levels tended to be negatively associated with the insulin‐stimulated glucose uptake after overfeeding. Therefore, the higher amino acid levels in LBW men could be a consequence of their reduction in skeletal muscle insulin sensitivity due to overfeeding with a possible increased skeletal muscle proteolysis and/or could potentially contribute to an impaired insulin sensitivity. Furthermore, the alanine level was negatively associated with the plasma acetylcarnitine level and positively associated with the hepatic glucose production after overfeeding. Thus, the higher alanine level in LBW men could be accompanied by an increased anaplerotic formation of oxaloacetate and thereby an enhanced tricarboxylic acid cycle activity and as well an increased gluconeogenesis.

## Introduction

Low birth weight (LBW) individuals have an increased risk of developing insulin resistance and type 2 diabetes later in life, compared with normal birth weight (NBW) individuals, when exposed to an affluent life style such as overfeeding (Hales et al. 1991; Barker et al. 1993; Hofman et al. 2004; Vaag et al. 2006). Indeed, otherwise healthy LBW men display several prediabetic metabolic abnormalities, including higher fasting blood glucose and serum insulin levels and a decreased hepatic insulin sensitivity compared with NBW men (Brons et al. 2008). Also, LBW in contrast to NBW men develop a decreased peripheral insulin sensitivity in response to a short‐term high‐fat, high‐calorie diet (Brons et al. 2012). Nevertheless, the underlying mechanisms behind the LBW prediabetic phenotype are not clear. Previously, we have found that LBW men exhibit an increased fatty acid oxidation along with a decreased glucose oxidation at night as determined during an isocaloric control diet and a high‐fat, high‐calorie diet compared with NBW men (Brons et al. 2013, 2015). Furthermore, LBW men have a higher relative contribution of fatty acid oxidation to the total energy expenditure at night and throughout 24 h when studied on the high‐fat, high‐calorie diet (Brons et al. 2015). Recently, we have extended our understanding of the LBW prediabetic phenotype with the finding of elevated fasting plasma acetylcarnitine levels in LBW men, pointing toward an increased acetyl‐CoA generation relative to its oxidation in the tricarboxylic acid (TCA) cycle (Ribel‐Madsen et al. 2016). An accumulation of acetyl‐CoA may theoretically lead to mitochondrial stress responses and the activation of serine kinases, which in turn may impair skeletal muscle insulin signaling and glucose transporter 4 (GLUT4) translocation (Muoio and Newgard 2008).

**Figure 1:**
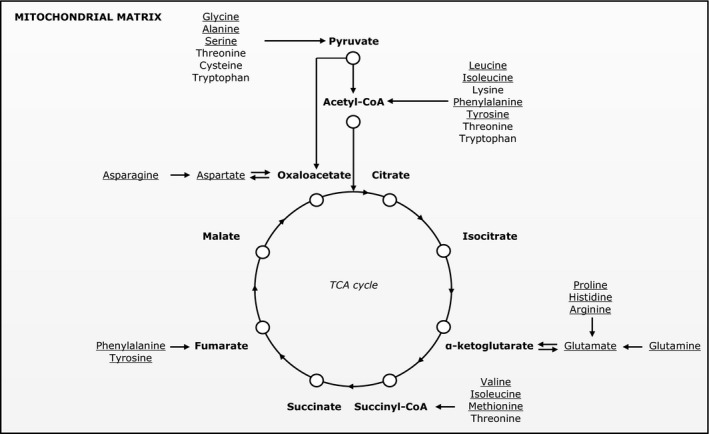
Amino acid anaplerotic and cataplerotic pathways that replenish or deplete tricarboxylic acid cycle intermediates, respectively. Plasma amino acid levels measured in this study are underlined.

In the present extension study, we hypothesized that the changes in fatty acid and glucose oxidation partitioning in LBW individuals would be associated with changes in plasma amino acid levels, reflecting the need for an adequate supply of TCA cycle intermediates to allow an efficient acetyl‐CoA oxidation in the TCA cycle (Fig. 1). Furthermore, such changes could be parts of the adverse metabolic events leading to insulin resistance in LBW individuals. In order to test our hypotheses, we measured fasting plasma levels of 15 individual or pools of amino acids in LBW and NBW men after an isocaloric control diet and after a 5‐day high‐fat, high‐calorie diet, and associated these levels to the plasma acetylcarnitine level, as a measure of the intracellular acetyl‐CoA level, and to measures of hepatic and peripheral insulin sensitivity.

## Materials and Methods

### Study population

Forty‐six young (23–27 years of age), healthy men were recruited from the Danish National Birth Registry according to birth weight. Among these, 20 individuals had LBW, which was defined as a birth weight within the 0–10th percentile range (2717 ± 268 g), as in a prior study (Jensen et al. 2002), and 26 individuals had NBW, which was defined as a birth weight within the 50–90th percentile range (3901 ± 207 g). All individuals were born at term (39–41 weeks of gestation) in Copenhagen in the period 1979–1980. Also, all participants were ensured to not have a family history of diabetes in two generations, not have a body mass index (BMI) greater than 30 kg/m^2^, not perform strenuous physical activity more than 10 h per week, not take pharmaceuticals that affect metabolism, and not have an abuse of alcohol or drugs.

### Study design

#### Diet interventions

**Figure 2:**
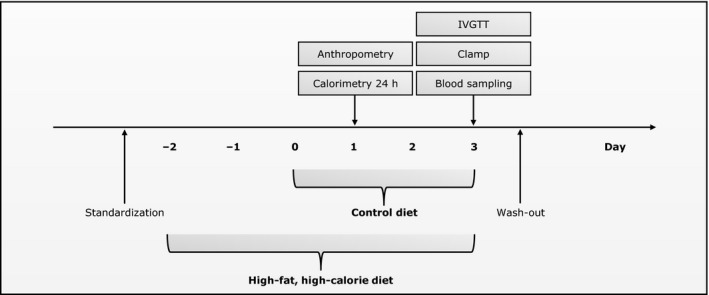
Study setup. Plasma amino acid levels were measured from blood samples collected following an overnight fast and immediately prior to the clamp examination on the last study day.

All individuals were in a randomized crossover setup standardized with respect to diet and physical activity and following given a 3‐day control diet and a 5‐day high‐fat, high‐calorie diet separated by a 6–8 weeks washout period (Fig. 2). Energy requirements of the individual subjects were calculated from a World Health Organization equation for men less than 30 years of age with a physical activity level of 1.4 corresponding to a low physical activity (WHO, 2001). The control diet was composed to replicate a habitual, weight‐maintaining diet (2819 ± 238 kcal/11,800 ± 1000 kJ) with 15% of the total energy from protein, 50% from carbohydrate, and 35% from fat, whereas the high‐fat, high‐calorie diet was prepared to contain 50% extra calories above the requirements (4228 ± 334 kcal/17,700 ± 1400 kJ) with 7.5% of the total energy from protein, 32.5% from carbohydrate, and 60% from fat (Table S1). Also, the meals contained in each intervention were identical from day to day. Dietary calculations were made in Dankost Pro (http://dankost.dk/english) (The National Food Agency, Copenhagen, Denmark).

#### Clinical examinations

Study activities were carried out over 3 days, with the first of these days being placed 1 or 3 days after the start of the control and high‐fat, high‐calorie diet intervention, respectively (Fig. 2). Anthropometry was performed on the first study day. An intravenous glucose tolerance test (IVGTT) and a hyperinsulinemic–euglycemic clamp were carried out in the morning on the third study day following an overnight fast to assess insulin secretion and sensitivity, as described previously (Brons et al. 2008, 2012). Furthermore, calorimetry was performed throughout 24 h from the first to second study day by use of a whole‐body respiratory chamber to evaluate substrate oxidation rates and energy expenditures, as described previously (Brons et al. 2013, 2015). Blood and tissue samples were collected prior to and during the clamp.

### Laboratory measurements

#### Amino acid analyses

Amino acid analyses were performed on EDTA‐plasma samples collected following an overnight fast (10.00 pm–7.00 am) and immediately prior to the clamp examination (Fig. 2). These analyses included a quantitative determination of 15 individual or pools of amino acids, noted in the tables by their three letter code, and were performed by use of sample preparation procedures and flow injection–tandem mass spectrometry, as described previously (Chace et al. 1995; Ferrara et al. 2008). In brief, plasma samples were spiked with known quantities of stable isotope‐labeled amino acid standards. Following, proteins were removed by precipitation with methanol and the supernatants were then evaporated to dryness under nitrogen gas and incubated with acidified butanol to form butyl ester derivatives of the amino acids. After this, the reagents were evaporated to dryness under nitrogen gas, and the samples were reconstituted in 1:1 (v/v) methanol:glycerol. Subsequently, the samples were introduced into a Quattro Micro MS/MS system (Waters, Milford, MA) equipped with a data system running MassLynx 4.0 (Waters). Mass spectra of the amino acid butyl esters were obtained by precursor ion and neutral loss scanning of [M+H]^+^ and [M+H–102]^+^ ions, respectively, the latter ion corresponding to a loss of butyl formate of 102 Da from the original [M+H]^+^ ion. Finally, amino acids were quantified from the ratio of their molecular signals to respective internal standards. Leucine and isoleucine were not resolved by the method, and therefore these amino acids are reported as a single analyte. Also, the quantification of these amino acids includes contributions from allo‐isoleucine and hydroxy‐proline, but these isobaric amino acids generally contribute little to the signal assigned to leucine and isoleucine (Chace et al. 1995). Furthermore, partial hydrolysis of asparagine to aspartic acid and of glutamine to glutamic acid occurs under the acidic conditions used to form butyl esters, and so the amounts of these amino acids plus their hydrolysis products are noted as Asx and Glx, respectively. Amino acid measurements were performed in The Sarah W. Stedman Nutrition and Metabolism Center Metabolomics/Biomarker Core Laboratory, Duke University, Durham, NC. The laboratory was blinded to the birth weight of the individuals.

#### Acylcarnitine analyses

Acylcarnitine analyses were performed on the same plasma samples as for the amino acid analyses. These analyses included a semiquantitative determination of 45 acylcarnitine species or sets of species and were performed as described previously (Ribel‐Madsen et al. 2016).

### Ethical approval

All study procedures were in accordance with the principles of the Declaration of Helsinki and were approved by the Regional Research Ethics Committee of Copenhagen, Denmark. Also, all participants were provided with written information on the study purpose and procedures and signed an informed consent prior to their participation.

### Statistical analyses

#### Amino acid levels and their relation to physiological measures

Differences in plasma amino acid levels between NBW and LBW individuals within each diet or between the control and high‐fat, high‐calorie diets within each birth weight group were assessed from Student's unpaired or paired *t*‐test (for normally distributed values), respectively, or Wilcoxon ranked‐sum or signed‐rank test (for not normally distributed values), respectively. Prior to these tests, statistical outliers (1.5 interquartile range) were removed from the dataset and replaced by the mean value within the given group. Normal distribution of the values (variables or differences between variables, respectively) was evaluated from Shapiro–Wilk test. Finally, adjustment for multiple testing was done by calculating false discovery rates, *Q*‐values, by the Benjamini and Hochberg method (Benjamini and Hochberg 1995). Data in Tables 2 and 4 are presented as mean value plus or minus standard deviation (SD) together with *P*‐ and *Q*‐values. *P* ≤ 0.05 were considered statistically significant if *Q* ≤ 0.2. Student's *t*‐test and Wilcoxon tests were performed in SAS Enterprise Guide 6.1 (SAS Institute, Cary, NC), and Benjamini and Hochberg corrections were performed in R 3.1.0 (https://www.r-project.org/).

Associations between individual plasma amino acid levels or between these levels and other variables were obtained from linear regression analyses. These analyses were performed on the pooled dataset of LBW and NBW individuals and were adjusted for age, BMI, and birth weight group. Data in Table 3 are presented as slope (plus or minus signs for a positive or negative association, respectively) and *P*‐value (number of plus or minus signs indicates the significance level) for those values meeting the false discovery rate criteria. *P*‐values were considered statistically significant as described above. Linear regression analyses and selected plots from these were performed in R.

## Results

Eighteen LBW and 25 NBW men were included in the present study. Two LBW men of the recruited participants failed to consume all the food during the high‐fat, high‐calorie diet, and a NBW subject felt discomfort during the clamp after the control diet and therefore did not further participate in this test in either the control or high‐fat, high‐calorie diet study part.

### Clinical characteristics

LBW and NBW men displayed differences in body composition and glucose and lipid metabolism after the control diet and high‐fat, high‐calorie diet, and both birth weight groups showed several changes in these parameters in response to the overfeeding challenge, as published previously (Brons et al. 2008, 2012, 2013, 2015; Ribel‐Madsen et al. 2016). A selection of these findings is presented in Tables 1 and S2.

**Table 1:** Clinical characteristics of low (LBW) and normal birth weight (NBW) men following the control (C) and high‐fat, high‐calorie (O) diets

	NBW			LBW			LBW versus NBW		
	(*n *= 25)			(*n *= 18)			(*n *= 18, *n *= 25)		
	C (mean ± SD)	O (mean ± SD)	*P*_NBW_	C (mean ± SD)	O (mean ± SD)	*P*_LBW_	*P*_C_	*P*_O_	*P*_Δ_
**Anthropometry**									
Birth weight (g)	3901 ± 207	**—**	**—**	2717 ± 268	**—**	**—**	**≤0.001**	**—**	**—**
Weight (kg)	78.4 ± 9.3	78.6 ± 9.7	n.s.	77.1 ± 11.3	77.1 ± 11.4	n.s.	n.s.	n.s.	n.s.
Height (m)	1.83 ± 0.07	**—**	**—**	1.77 ± 0.05	**—**	**—**	**≤0.05**	**—**	**—**
Body mass index (kg/m^2^)	23.3 ± 2.4	23.3 ± 2.5	n.s.	24.6 ± 3.8	24.6 ± 3.8	n.s.	n.s.	n.s.	n.s.
**Clamp**									
*Basal*									
B‐Glucose (mmol/L)	4.59 ± 0.47	5.05 ± 0.40	**≤0.001**	4.97 ± 0.48	5.18 ± 0.34	**≤0.05**	**≤0.01**	n.s.	n.s.
S‐Insulin (pmol/L)	30.2 ± 14.7	43.4 ± 29.2	**≤0.05**	41.7 ± 14.6	44.7 ± 21.9	n.s.	**≤0.01**	n.s.	n.s.
P‐NEFA (μmol/L)	334 ± 136	205 ± 82	**≤0.001**	406 ± 200	188 ± 91	**≤0.001**	n.s.	n.s.	n.s.
P‐Acetylcarnitine (μmol/L)	4.771 ± 0.797	3.985 ± 0.738	**≤0.01**	5.985 ± 1.587	4.393 ± 0.784	**≤0.001**	**≤0.01**	n.s.	n.s.
HGP (mg/kg·FFM/min)	2.21 ± 0.48	2.85 ± 0.99	**≤0.01**	2.40 ± 0.5	2.48 ± 0.5	n.s.	n.s.	n.s.	**≤0.05**
Hepatic IR (mg/kg·FFM/min·pmol/L)	68.7 ± 34.1	113.7 ± 61.5	**≤0.001**	102.3 ± 50.8	108.7 ± 55.5	n.s.	**≤0.05**	n.s.	**≤0.05**
*Insulin‐stimulated*									
P‐NEFA (μmol/L)	9.29 ± 4.39	12.42 ± 6.43	**≤0.01**	9.56 ± 5.03	14.39 ± 7.76	**≤0.01**	n.s.	n.s.	n.s.
M‐value (mg/kg·FFM/min)	13.73 ± 2.32	13.29 ± 3.32	n.s.	13.47 ± 3.14	11.89 ± 3.57	**≤0.05**	n.s.	n.s.	n.s.
**IVGTT**									
FPIR (pmol/L)	1894 ± 1431	2604 ± 1793	**≤0.001**	2135 ± 1034	2750 ± 1509	**≤0.01**	n.s.	n.s.	n.s.
Hepatic DI	0.38 ± 0.63	0.25 ± 0.21	n.s.	0.21 ± 0.11	0.24 ± 0.13	n.s.	n.s.	n.s.	n.s.
Peripheral DI	0.29 ± 0.19	0.35 ± 0.20	**≤0.05**	0.33 ± 0.13	0.32 ± 0.17	n.s.	n.s.	n.s.	n.s.

Data are presented as mean ± SD. *P*‐values from Student's *t*‐test are presented unadjusted for multiple comparisons, and *P* ≤ 0.05 are considered statistically significant. *P*_NBW_ and *P*_LBW_: O versus C diet within each birth weight group, *P*_C_ and *P*_O_: LBW versus NBW individuals within each diet, *P*_Δ_: LBW versus NBW individuals on response values. n.s.: Not significant. *P* ≤ 0.05 are marked in bold.

Abbreviations: B, blood; DI, disposition index; FFM, fat‐free mass; FPIR, first‐phase insulin response; HGP, hepatic glucose production; IR, insulin resistance; IVGTT, intravenous glucose tolerance test; NEFA, nonesterified fatty acid; P, plasma; S, serum.

### Amino acid levels and their relation to physiological measures

LBW and NBW men only displayed tendencies to differences in plasma amino acid levels after the control diet when accounted for multiple testing, but significant differences after the high‐fat, high‐calorie diet, and both groups showed several changes in amino acid levels in response to overfeeding (Table 2).

**Table 2:** Plasma amino acid levels in low (LBW) and normal birth weight (NBW) men following the control (C) and high‐fat, high‐calorie (O) diets

(μmol/L)	NBW			LBW			LBW versus NBW		
	(*n *= 25)			(*n *= 18)			(*n *= 18, *n *= 25)		
	C (mean ± SD)	O (mean ± SD)	*P*_NBW_ *Q*_NBW_	C (mean ± SD)	O (mean ± SD)	*P*_LBW_ *Q*_LBW_	*P*_C_ *Q*_C_	*P*_O_ *Q*_O_	*P*_Δ_ *Q*_Δ_
**Amino acid profiling**									
Gly	320.6 ± 36.0	319.1 ± 35.0	0.8304	319.6 ± 30.4	320.2 ± 38.4	0.9531	0.9205	0.9512	0.8618
Ala	286.5 ± 58.3	345.0 ± 53.3	**0.0007** **0.0021**	288.6 ± 78.0	403.9 ± 87.5	**<0.0001** **0.0008**	0.8929	**0.0174** **0.1305**	**0.0134** 0.2010
Ser	109.9 ± 16.2	104.3 ± 14.6	0.1590	100.2 ± 13.5	106.7 ± 10.9	0.0673	**0.0445** 0.2455	0.5590	**0.0291** 0.2110
Pro	157.2 ± 19.2	145.0 ± 24.6	**0.0007** **0.0021**	180.0 ± 42.8	169.2 ± 42.4	0.1183	**0.0462** 0.2455	**0.0396** **0.1485**	0.8532
Val	234.4 ± 30.2	205.9 ± 11.0	**<0.0001** **0.0005**	238.0 ± 33.4	216.4 ± 25.9	**0.0258** **0.0774**	0.7138	0.1185	0.4983
Leu/Ile	178.6 ± 24.5	146.0 ± 16.6	**<0.0001** **0.0005**	181.0 ± 19.5	152.9 ± 13.4	**<0.0001** **0.0008**	0.7359	0.1527	0.4522
Met	27.1 ± 3.7	25.5 ± 3.2	**0.0358** **0.0761**	27.8 ± 2.4	28.2 ± 3.7	0.5893	0.4757	**0.0135** **0.1305**	0.0625
His	65.1 ± 6.6	58.4 ± 5.6	**<0.0001** **0.0005**	62.3 ± 7.9	58.8 ± 5.7	0.1042	0.2157	0.8456	0.1920
Phe	60.3 ± 7.8	58.1 ± 7.8	0.1171	60.9 ± 5.2	57.2 ± 6.6	**0.0065** **0.0244**	0.3364	0.8929	0.7413
Tyr	53.7 ± 7.0	57.5 ± 9.3	**0.0406** **0.0761**	58.1 ± 7.2	61.9 ± 10.7	0.1622	**0.0491** 0.2455	0.1583	0.9917
Asx	144.0 ± 20.8	136.1 ± 24.0	0.2760	148.2 ± 23.7	149.4 ± 24.7	0.9006	0.5486	0.0853	0.4351
Glx	56.1 ± 11.0	63.6 ± 15.8	**0.0035** **0.0088**	63.1 ± 13.6	71.5 ± 19.9	0.1118	0.0687	0.1513	0.6688
Orn	58.4 ± 6.6	61.5 ± 12.1	0.2189	59.3 ± 10.6	58.6 ± 8.6	0.8457	0.7437	0.3972	0.3657
Cit	28.1 ± 4.0	28.8 ± 5.5	0.5057	29.6 ± 3.5	32.6 ± 5.2	**0.0065** **0.0244**	0.1997	**0.0284** **0.1420**	0.1463
Arg	90.0 ± 14.9	85.2 ± 13.1	0.0875	90.4 ± 12.3	92.7 ± 13.5	0.4444	0.9296	0.0763	0.0601
Total levels									
Essential	655.5 ± 67.8	579.2 ± 33.2	**<0.0001**	660.4 ± 53.8	606.2 ± 43.7	**0.0035**	0.8006	**0.0260**	0.2603
Nonessential	1128 ± 101.1	1171 ± 107.5	0.0980	1158 ± 98.4	1283 ± 131.8	**0.0004**	0.3418	**0.0038**	**0.0353**
All	1870 ± 157.1	1840 ± 131.9	0.3896	1907 ± 132.7	1980 ± 159.4	0.0805	0.4205	**0.0030**	0.0556

Data are presented as mean ± SD. *P* ≤ 0.05 are presented together with *Q*‐values, and *P* ≤ 0.05 with corresponding *Q* ≤ 0.2 are considered statistically significant. *P*_NBW_ and *P*_LBW_: O versus C diet within each birth weight group, *P*_C_ and *P*_O_: LBW versus NBW individuals within each diet, *P*_Δ_: LBW versus NBW individuals on response values. *P* ≤ 0.05 and *Q* ≤ 0.2 are marked in bold. Essential amino acids: Val, Leu/Ile, Met, His, Phe, and Arg; nonessential amino acids: Gly, Ala, Ser, Pro, Tyr, Asx, and Glx.

LBW men tended to have higher proline and tyrosine levels and a lower serine level after the control diet compared with NBW men. Furthermore, LBW and NBW men both increased alanine levels and decreased valine, leucine/isoleucine, and essential amino acid levels in response to overfeeding. In addition, LBW men increased citrulline and nonessential amino acid levels and decreased the phenylalanine level due to overfeeding, whereas NBW men increased tyrosine and glutamine/glutamic acid levels and decreased proline, methionine, and histidine levels in response to this challenge. Also, LBW men had higher alanine, proline, methionine, and citrulline levels after the high‐fat, high‐calorie diet compared with NBW men, and as well higher essential, nonessential, and total amino acid levels after this diet.

**Figure 3:**
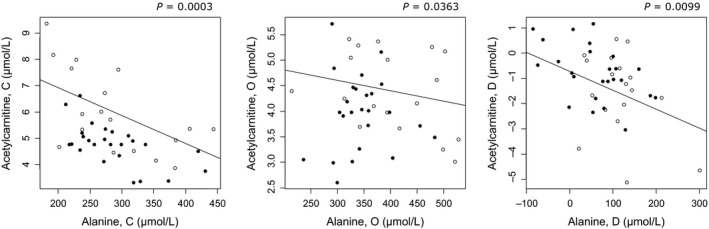
Associations between plasma alanine and acetylcarnitine levels following the control (C) and high‐fat, high‐calorie (O) diets and between response values (D). Open circles: Low birth weight (LBW) individuals, filled circles: Normal birth weight (NBW) individuals.

Plasma alanine levels were negatively associated with the fasting plasma acetylcarnitine level after both the control diet and high‐fat, high‐calorie diet (Fig. 3). Furthermore, an increase in the alanine level due to overfeeding was associated with a decrease in the acetylcarnitine level (Fig. 3). In addition, both alanine and total amino acid levels tended to be negatively associated with the insulin‐stimulated glucose uptake rate, M‐value, (*P *= 0.0911, *Q *= 0.1707; *P *= 0.0846, *Q *= 0.2115, respectively) after the high‐fat, high‐calorie diet (Table 3). Also, an increase in the proline level was associated with a decrease in the M‐value (*P *= 0.0271, *Q *= 0.1350) (Table 3). Furthermore, alanine and total amino acid levels were positively associated with the hepatic glucose production (*P *= 0.0022, *Q *= 0.0110; *P *= 0.0114, *Q *= 0.0570, respectively) after the high‐fat, high‐calorie diet (Table 3).

**Table 3:** Associations between plasma amino acid levels and physiological measures following the control (C) and high‐fat, high‐calorie (O) diets and between response values (Δ)

		Gly	Ala	Ser	Pro	Val	Leu/ Ile	Met	His	Phe	Tyr	Asx	Glx	Orn	Cit	Arg	All
**Clamp**																	
*Basal*																	
B‐Glucose	C															+	
	O																
	Δ			−−													
S‐Insulin	C												(+)				
	O							−−								−	
	Δ																
HGP	C												+ +				
	O		+ +			(+)											+
	Δ									+							
Hepatic IR	C												+				
	O							(−)									
	Δ																
*Insulin‐stimulated*																	
M‐value	C	+														(−)	
	O		(−)			(−)											
	Δ				−												

Data are presented as slope (+/− for a positive or negative association, respectively) and *P*‐value (+/−: *P* ≤ 0.05, + +/− −: *P* ≤ 0.01, and (+)/(−): *P* ≤ 0.1) for those values meeting the false discovery rate criteria. *P* ≤ 0.05 with corresponding *Q* ≤ 0.2 are considered statistically significant. Regression analyses were performed on the pooled dataset of low (LBW) and normal birth weight (NBW) individuals and were adjusted for age, BMI, and birth weight group.

Abbreviations: See Table 1.

## Discussion

We demonstrated that LBW men had higher fasting plasma alanine, proline, methionine, citrulline, and total amino acid levels after the high‐fat, high‐calorie diet compared with NBW men. Furthermore, the plasma alanine level was negatively associated with the fasting plasma acetylcarnitine level, which reflects the intracellular acetyl‐CoA pool (Adams et al. 2009), after both the control diet and high‐fat, high‐calorie diet. Previously, we have shown that LBW men have a higher plasma acetylcarnitine level after the control diet and a tendency to this after the high‐fat, high‐calorie diet compared with NBW men (Ribel‐Madsen et al. 2016), and in addition, an increased fatty acid oxidation and a decreased glucose oxidation at night during intake of both diets (Brons et al. 2013, 2015). Taken together, we proposed that the acetyl‐CoA generation from in particular the increased fatty acid oxidation exceeds its oxidation in the TCA cycle in LBW individuals (Ribel‐Madsen et al. 2016), possibly due to a depletion of TCA cycle intermediates. In the state of a high intracellular acetyl‐CoA concentration, as seems to be the case in LBW individuals (Ribel‐Madsen et al. 2016), pyruvate dehydrogenase is inhibited and pyruvate carboxylase is activated, favoring pyruvate carboxylation to oxaloacetate, the four‐carbon unit TCA cycle intermediate that condenses with acyl‐CoA to form citrate, and as well favoring pyruvate transamination to alanine (Hue and Taegtmeyer 2009). This promotes anaplerosis and gluconeogenesis, respectively (Hue and Taegtmeyer 2009). Therefore, the higher plasma alanine level in LBW men could reflect an increased pyruvate transamination to alanine in tissues, and the negative association between the plasma alanine and acetylcarnitine levels may be due to an increased anaplerotic formation of oxaloacetate. A recent study has reported that induced pluripotent stem cells from patients with genetic insulin resistance have a reduced mitochondrial oxidative function, including a decreased citrate synthase activity (Burkart et al. 2016). Also, addition of exogenous oxaloacetate to the cell culture system could resolve citrate synthase activity, and it was therefore proposed that substrate availability could be a limiting factor of TCA cycle activity in insulin resistance (Burkart et al. 2016). Alternatively, the higher plasma alanine level in LBW men could be a result of an increased skeletal muscle proteolysis and de novo alanine synthesis, as further discussed below. Indeed, LBW men display a higher adjusted total energy expenditure at night during the high‐fat, high‐calorie diet compared with NBW men (Brons et al. 2015). Therefore, the TCA cycle is likely to be upregulated in LBW individuals at night, and we propose that a higher plasma alanine level in these individuals could reflect an increased availability of alanine and pyruvate in tissues for anaplerotic formation of oxaloacetate and furthermore that this may enhance the acetyl‐CoA oxidation in the TCA cycle, which in turn may contribute to development of hepatic insulin resistance (Satapati et al. 2012).

We furthermore demonstrated that plasma alanine and total amino acid levels tended to be negatively associated with the insulin‐stimulated glucose uptake rate, M‐value, after the high‐fat, high‐calorie diet. Also, we have previously demonstrated that LBW men decreased the M‐value in response to this diet (Brons et al. 2012). Whether the higher plasma alanine and total amino acid levels in LBW individuals could be a consequence of and/or contribute to impaired skeletal muscle insulin sensitivity is uncertain. Insulin has a suppressive effect on tissue proteolysis, and so an impaired skeletal muscle insulin sensitivity may increase the amino acid release from skeletal muscle (Magkos et al. 2010). Also, the amino acid release from skeletal muscle following an overnight fast does not reflect its amino acid composition (Ruderman 1975), and so relatively more alanine and glutamine, which represent the main amino acid gluconeogenic precursors in liver and kidney, respectively, (Felig 1973; Felig et al. 1977; Stumvoll et al. 1999), are released (Ruderman 1975; Felig et al. 1977). Actually, alanine and glutamine together accounts for approximately two thirds of the amino acids released from skeletal muscle (Ruderman 1975; Garber et al. 1976). This has been suggested to be due to an in situ amino acid metabolism that results in de novo synthesis of nonessential amino acids, primarily alanine and glutamine (Ruderman 1975; Felig et al. 1977). Alanine is generated through pyruvate transamination (Ruderman 1975), and it has been suggested that pyruvate to this synthesis may be derived from other amino acids, as skeletal muscle theoretically could generate pyruvate from glutamate, aspartate, and other amino acids that are metabolized in the TCA cycle (Ruderman 1975). Notably, it has been demonstrated that more alanine relative to glutamine is released from skeletal muscle in subjects with diabetes (Jungas et al. 1992). It is therefore interesting that LBW men showed a tendency to a larger relative contribution of alanine and nonessential amino acids to the total plasma amino acid level after the high‐fat, high‐calorie diet compared with NBW men (Table 4). Accordingly, the higher plasma alanine level in LBW individuals could be a result of both an increased skeletal muscle proteolysis due to an impaired skeletal muscle insulin sensitivity and of an increased de novo alanine synthesis from other amino acids, including in particular glutamate, aspartate, valine, leucine, and isoleucine, and pyruvate (Ruderman 1975; Snell 1980). Although this is expected to cause reductions in the relative contributions of these amino acids to the total plasma amino acid level in LBW men, we did not observe this (Table 4). As concerns, the possibility that the higher plasma amino acid levels in LBW individuals could contribute to an impaired skeletal muscle insulin sensitivity, it has been shown that short‐term elevation of plasma amino acid levels to postprandial concentrations causes insulin resistance by direct inhibition of muscle glucose transport and/or phosphorylation with a subsequent reduction in glycogen synthesis rates (Krebs et al. 2002). In addition to the potential effects of amino acids on skeletal muscle insulin sensitivity, we demonstrated that plasma alanine and total amino acid levels were positively associated with the hepatic glucose production after the high‐fat, high‐calorie diet. Higher plasma amino acid levels could induce this effect on the liver via indirect and/or direct mechanisms. Thus, amino acids can stimulate insulin and glucagon secretion (Floyd et al. 1966; Ohneda et al. 1968; Newsholme et al. 2006), and changes in the portal vein concentration of these hormones could increase the endogenous glucose production (Roden et al. 1996). On the other hand, amino acids could by acting as substrates induce gluconeogenesis and thereby enhance the endogenous glucose production (Rui 2014). It is notable that the plasma glutamine/glutamic acid and alanine levels were strongly positively associated with the hepatic glucose production after the control or high‐fat, high‐calorie diet, respectively, (Table 3), keeping in mind that these amino acids are the main amino acid gluconeogenic precursors (Felig 1973; Felig et al. 1977; Stumvoll et al. 1999). Previously, we have shown that LBW men have a higher fasting blood glucose level after the control diet compared with NBW men (Brons et al. 2008), and we propose that an increased gluconeogenesis, occurring parallel to an increased hepatic fatty acid oxidation, may contribute to this. Alanine and glutamine metabolism in the liver for gluconeogenesis results in the production of ammonia. Therefore, the higher plasma citrulline level in LBW men after the high‐fat, high‐calorie diet could reflect an increased metabolism of these amino acids with a subsequent increased urea cycle activity due to a greater demand of ammonia removal.

**Table 4:** Relative plasma amino acid levels compared to the total plasma amino acid level in low (LBW) and normal birth weight (NBW) men following the control (C) and high‐fat, high‐calorie (O) diets

(Fraction)	NBW			LBW			LBW versus NBW		
	(*n *= 25)			(*n *= 18)			(*n *= 18, *n *= 25)		
	C (mean ± SD)	O (mean ± SD)	*P*_NBW_ *Q*_NBW_	C (mean ± SD)	O (mean ± SD)	*P*_LBW_ *Q*_LBW_	*P*_C_ *Q*_C_	*P*_O_ *Q*_O_	*P*_Δ_ *Q*_Δ_
**Amino acid profiling**									
Rel Gly	0.172 ± 0.017	0.174 ± 0.016	0.6024	0.168 ± 0.017	0.162 ± 0.018	0.1692	0.4775	**0.0306** **0.1759**	0.1473
Rel Ala	0.152 ± 0.021	0.187 ± 0.019	**<0.0001** **0.0005**	0.150 ± 0.035	0.202 ± 0.033	**<0.0001** **0.0005**	0.8284	0.0607	0.0569
Rel Ser	0.059 ± 0.008	0.057 ± 0.006	0.2302	0.053 ± 0.007	0.054 ± 0.005	0.3978	**0.0105** **0.1575**	0.1345	0.1614
Rel Pro	0.084 ± 0.009	0.079 ± 0.011	**0.0007** **0.0021**	0.094 ± 0.021	0.085 ± 0.018	**0.0019** **0.0057**	**0.0385** 0.2888	0.1500	0.2023
Rel Val	0.125 ± 0.012	0.112 ± 0.010	**<0.0001** **0.0005**	0.125 ± 0.014	0.109 ± 0.011	**0.0003** **0.0011**	0.8817	0.3412	0.5603
Rel Leu/Ile	0.095 ± 0.008	0.079 ± 0.008	**<0.0001** **0.0005**	0.095 ± 0.008	0.077 ± 0.005	**<0.0001** **0.0005**	0.8682	0.3504	0.5328
Rel Met	0.014 ± 0.002	0.014 ± 0.001	**0.0489** **0.0917**	0.015 ± 0.001	0.014 ± 0.002	0.3376	0.8000	0.4082	0.5281
Rel His	0.035 ± 0.004	0.032 ± 0.002	**0.0002** **0.0008**	0.033 ± 0.005	0.030 ± 0.004	**0.0186** **0.0465**	0.0957	**0.0428** **0.1759**	0.8346
Rel Phe	0.032 ± 0.003	0.032 ± 0.003	0.1942	0.032 ± 0.003	0.029 ± 0.003	**<0.0001** **0.0005**	0.8210	**0.0100** **0.1500**	**0.0031** **0.0465**
Rel Tyr	0.029 ± 0.003	0.031 ± 0.004	**0.0052** **0.0130**	0.030 ± 0.003	0.031 ± 0.004	0.4836	0.0861	0.9512	0.1441
Rel Asx	0.077 ± 0.011	0.074 ± 0.013	0.3956	0.078 ± 0.012	0.076 ± 0.013	0.6593	0.8953	0.6937	0.8523
Rel Glx	0.030 ± 0.005	0.034 ± 0.008	**0.0064** **0.0137**	0.033 ± 0.007	0.037 ± 0.011	0.1960	0.1126	0.4800	0.6973
Rel Orn	0.031 ± 0.004	0.033 ± 0.006	0.1522	0.031 ± 0.006	0.030 ± 0.005	0.4918	0.8797	**0.0469** **0.1759**	0.1479
Rel Cit	0.015 ± 0.002	0.016 ± 0.003	0.1384	0.016 ± 0.002	0.017 ± 0.003	0.0790	0.3957	0.3707	0.6564
Rel Arg	0.048 ± 0.007	0.046 ± 0.007	0.1866	0.048 ± 0.007	0.047 ± 0.007	0.6541	0.7784	0.7904	0.5188
Total levels									
Rel Essential	0.350 ± 0.018	0.315 ± 0.017	**<0.0001**	0.347 ± 0.021	0.307 ± 0.018	**<0.0001**	0.5284	0.1148	0.4509
Rel Nonessential	0.603 ± 0.018	0.635 ± 0.017	**<0.0001**	0.607 ± 0.020	0.647 ± 0.021	**<0.0001**	0.5464	0.0576	0.2379

Data are presented as mean ± SD. *P* ≤ 0.05 are presented together with *Q*‐values, and *P* ≤ 0.05 with corresponding *Q* ≤ 0.2 are considered statistically significant. *P*_NBW_ and *P*_LBW_: O versus C diet within each birth weight group, *P*_C_ and *P*_O_: LBW versus NBW individuals within each diet, *P*_Δ_: LBW versus NBW individuals on response values. *P* ≤ 0.05 and *Q* ≤ 0.2 are marked in bold. Essential amino acids: Val, Leu/Ile, Met, His, Phe, and Arg; nonessential amino acids: Gly, Ala, Ser, Pro, Tyr, Asx, and Glx.

In similarity to our study, higher plasma amino acid levels have repeatedly been reported to associate with insulin resistance (Newgard et al. 2009; Tai et al. 2010; Wurtz et al. 2012a; Nakamura et al. 2014; Seibert et al. 2015; Pedersen et al. 2016). To this end, plasma amino acid levels, in particular of the branched chain amino acids valine, leucine, and isoleucine, aromatic amino acids tyrosine and phenylalanine, and alanine, are predictive of development of type 2 diabetes even several years before its onset (Wang et al. 2011; Wurtz et al. 2012b, 2013; Yamakado et al. 2015). Importantly, higher fasting serum valine, leucine, isoleucine, and phenylalanine levels have been shown to predict increased fasting and 2 h postchallenge blood glucose levels after a 6.5‐year follow‐up (Wurtz et al. 2012b), whereas higher levels of gluconeogenic precursors, including alanine, lactate, and pyruvate, predict increased 2 h postchallenge, but not fasting blood glucose levels, after this follow‐up (Wurtz et al. 2012b). Therefore, it was concluded that gluconeogenic precursors could be potential markers of long‐term impaired insulin sensitivity that may relate to attenuated glucose tolerance later in life (Wurtz et al. 2012b). Another study performed in rodent has described that an increased plasma citrulline level may predict development of the metabolic syndrome (Sailer et al. 2013). Thus, our findings of elevated plasma amino acid levels, including alanine and citrulline levels, in LBW men exposed to a high‐fat, high‐calorie diet challenge reveal additional metabolic abnormalities in these individuals associated to early stages of development of insulin resistance and type 2 diabetes.

**Figure 4:**
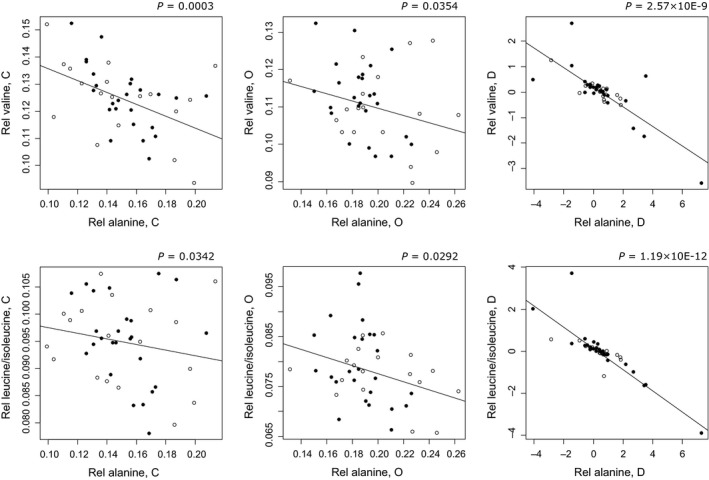
Associations between relative plasma alanine and valine or leucine/isoleucine levels, respectively, following the control (C) and high‐fat, high‐calorie (O) diets and between response values (D). Open circles: Low birth weight (LBW) individuals, filled circles: Normal birth weight (NBW) individuals.

We, moreover, demonstrated that LBW and NBW men both increased the plasma alanine level and decreased valine and leucine/isoleucine levels in response to overfeeding. Furthermore, an increase in the plasma alanine level was associated with a decrease in the fasting plasma acetylcarnitine level. Therefore, an increase in the plasma alanine level due to overfeeding could be accompanied by an increase in the formation of oxaloacetate that may enhance the acetyl‐CoA oxidation. Interestingly, LBW men showed a tendency to a larger increase in the plasma alanine level in response to overfeeding compared with NBW men (Table 2), and in addition, a tendency to a larger increase in the relative contribution of alanine to the total plasma amino acid level (Table 4). This could be due to an increased skeletal muscle alanine release as a consequence of their decline in skeletal muscle insulin sensitivity during overfeeding (Brons et al. 2012). Valine, leucine, and isoleucine are the major nitrogen sources for de novo alanine synthesis in skeletal muscle (Felig et al. 1977; Haymond and Miles 1982). Thus, the decrease in plasma valine and leucine/isoleucine levels in LBW and NBW men in response to overfeeding could indicate an increased metabolism of these amino acids to alanine in skeletal muscle. A decrease in these amino acid levels as well as in other essential amino acid levels could also be a result of the lower protein content in the high‐fat, high‐calorie diet compared to the control diet (Table S1). However, it is remarkable that an increase in the relative plasma alanine level was strongly significantly associated with decreases in both relative plasma valine and leucine/isoleucine levels (Fig. 4), indicating that an increased metabolism of these amino acids to alanine could take place. LBW men additionally showed a tendency to a larger increase in the serine level in response to overfeeding compared with NBW men (Table 2). Serine is, together with alanine, a precursor to pyruvate, and so an increased availability of this amino acid may contribute to an increased anaplerosis and thus an enhanced TCA cycle activity as well as to an increased gluconeogenesis.

Our study is the first to investigate fasting plasma amino acid levels in LBW individuals susceptible of developing type 2 diabetes after a control diet and after a high‐fat, high‐calorie diet. It has its strengths in the careful selection of LBW and NBW subjects, highly standardized study setup, and thorough physiological and metabolic characterization of the individuals. However, in relation to the biological interpretation of the results, and especially of the association between the amino acid availability and acetyl‐CoA oxidation in the TCA cycle, it has its limitations in the metabolite profiling being restricted to plasma amino acids and acylcarnitines, and thereby not having pyruvate and TCA cycle intermediates in its range. Furthermore, skeletal muscle biopsies from the LBW and NBW men collected prior to and during the clamp examination are snap frozen, which limits the possibilities of performing supplementary functional studies, including for instance measurements of the cellular localization of GLUT4 (Ploug et al. 1998; Lauritzen et al. 2008). In conclusion, our study describes elevated fasting plasma amino acid levels in LBW men after a 5‐day high‐fat, high‐calorie diet, including alanine, proline, methionine, citrulline, and total amino acid levels. Furthermore, these elevated plasma amino acid levels, and in particular of alanine, may be a result of an impaired glucose oxidation and/or an increased skeletal muscle proteolysis, and could be part of the adverse metabolic changes leading to skeletal muscle and hepatic insulin resistance in LBW individuals.

## Conflicts of Interest

All authors declare no financial or otherwise conflicts of interest in this study.

## Supporting information




**Table S1**. Protein, carbohydrate, and fat contents of the control (C) and high‐fat, high‐calorie (O) diets.
**Table S2.** Glucose, fatty acid, and protein oxidation rates and total energy expenditures in low (LBW) and normal birth weight (NBW) men during the control (C) and high‐fat, high‐calorie (O) diets.
