# Supplementary material for: Plasma amino acid levels are elevated in young, healthy low birth weight men exposed to short‐term high‐fat overfeeding
Source: Physiol Rep. 2016 Dec 12;4(23):e13044. doi: 10.14814/phy2.13044 (PMC5260087; doi:10.14814/phy2.13044)
Supplement: Supplementary file 1 — Table S1. Protein, carbohydrate, and fat contents of the control (C) and high‐fat, high‐calorie (O) diets. Table S2. Glucose, fatty acid, and protein oxidation rates and total energy expenditures in low (LBW) and normal birth weight (NBW) men during the control (C) and high‐fat, high‐calorie (O) diets. [file PHY2-4-13044-s001.docx]

**SUPPORTING INFORMATION**

**Table S1**: **Protein, carbohydrate, and fat contents of the control (C) and high-fat, high-calorie (O) diets.**

|  | **C** | | **O** | | **O/C** | |
| --- | --- | --- | --- | --- | --- | --- |
|  | **Total**  (Mean) | **Per 100 g**  (Mean) | **Total**  (Mean) | **Per 100 g**  (Mean) | **Total**  (Ratio) | **Per 100 g**  (Ratio) |
| **Energy (kJ)** |  |  |  |  |  |  |
| Total | 9664 | 698 | 14848 | 1135 | 1.54 | 1.63 |
| **Energy (E%)** |  |  |  |  |  |  |
| Protein | 15 | 15 | 8 | 8 | 0.53 | 0.53 |
| Carbohydrate | 49 | 49 | 33 | 33 | 0.67 | 0.67 |
| Fat | 35 | 35 | 60 | 60 | 1.71 | 1.71 |
| **Energy (g)** |  |  |  |  |  |  |
| Protein | 88 | 6.4 | 67.1 | 5.1 | 0.76 | 0.80 |
| Carbohydrate | 266.7 | 19.2 | 277.6 | 21.2 | 1.04 | 1.10 |
| Fat | 92.1 | 6.6 | 239 | 18.3 | 2.60 | 2.77 |
| **Fat (g)** |  |  |  |  |  |  |
| Saturated fatty acids | 35.6 | 2.6 | 109.8 | 8.4 | 3.08 | 3.23 |
| Monounsaturated fatty acids | 31.5 | 2.3 | 85.4 | 6.5 | 2.71 | 2.83 |
| Polyunsaturated fatty acids | 8.6 | 0.6 | 28.5 | 2.2 | 3.31 | 3.67 |
| n-3 fatty acids | 0.9 | 0.1 | 5.8 | 0.4 | 6.44 | 4.00 |
| n-6 fatty acids | 7.2 | 0.5 | 21.8 | 1.7 | 3.03 | 3.40 |

**Table S2:** **Glucose, fatty acid, and protein oxidation rates and total energy expenditures in low (LBW) and normal birth weight (NBW) men during the control (C) and high-fat, high-calorie (O) diets.** Data are presented as mean ± SEM. *P*-values from Student’s *t*-tests are presented unadjusted for multiple comparisons, and *P*-values ≤0.05 are considered statistically significant. *P*_NBW_ and *P*_LBW_: O versus C diet within each birth weight group, *P*_C_ and *P*_O_: LBW versus NBW individuals within each diet, *P*_Δ_: LBW versus NBW individuals on response values. *P*-values ≤0.05 are marked in bold. Details on the measurements have been described in previous articles (Brons *et al.*, 2013; Brons *et al.*, 2015). Abbreviations: EE, Energy expenditure; FOX, Fatty acid oxidation; GOX, Glucose oxidation; POX, Protein oxidation.

|  | **NBW**  (*n* = 26) | | | **LBW**  (C: *n* = 20, O: *n* = 18) | | | **LBW versus NBW**  (*n* = 20/*n* = 18, *n* = 26) | | |
| --- | --- | --- | --- | --- | --- | --- | --- | --- | --- |
| (kJ/min) | **C**  (Mean ± SEM) | **O**  (Mean ± SEM) | ***P*_NBW_** | **C**  (Mean ± SEM) | **O**  (Mean ± SEM) | ***P*_LBW_** | ***P*_C_** | ***P*_O_** | ***P*_Δ_** |
| **Calorimetry 24 h** |  |  |  |  |  |  |  |  |  |
| **GOX** |  |  |  |  |  |  |  |  |  |
| Day | 3.85 ± 0.17 | 3.50 ± 0.08 | **0.0297** | 3.69 ± 0.16 | 3.30 ± 0.14 | 0.0609 | 0.52 | 0.19 | 0.94 |
| Night | 1.97 ± 0.10 | 2.07 ± 0.07 | 0.3126 | 1.78 ± 0.09 | 1.84 ± 0.10 | 0.3391 | 0.18 | 0.06 | 0.97 |
| Sleep | 1.91 ± 0.12 | 1.89 ± 0.08 | 0.9131 | 1.58 ± 0.10 | 1.77 ± 0.11 | 0.0836 | **0.05** | 0.37 | 0.21 |
| 24 h | 3.10 ± 0.13 | 2.93 ± 0.07 | 0.1510 | 2.92 ± 0.13 | 2.73 ± 0.09 | 0.2620 | 0.34 | 0.09 | 0.97 |
| **FOX** |  |  |  |  |  |  |  |  |  |
| Day | 3.34 ± 0.16 | 4.23 ± 0.14 | **<0.0001** | 3.46 ± 0.14 | 4.52 ± 0.21 | **<0.0001** | 0.60 | 0.23 | 0.60 |
| Night | 2.34 ± 0.10 | 2.80 ± 0.10 | **0.0005** | 2.60 ± 0.08 | 3.06 ± 0.12 | **0.0023** | 0.07 | 0.10 | 0.93 |
| Sleep | 2.14 ± 0.14 | 2.72 ± 0.12 | **0.0001** | 2.50 ± 0.09 | 2.87 ± 0.13 | **0.0221** | **0.05** | 0.38 | 0.40 |
| 24 h | 2.92 ± 0.12 | 3.63 ± 0.12 | **<0.0001** | 3.11 ± 0.11 | 3.91 ± 0.14 | **<0.0001** | 0.24 | 0.14 | 0.76 |
| **POX** |  |  |  |  |  |  |  |  |  |
| Day | 1.13 ± 0.04 | 0.79 ± 0.03 | **<0.0001** | 1.08 ± 0.04 | 0.74 ± 0.04 | **<0.0001** | 0.48 | 0.32 | 0.71 |
| Night | 1.13 ± 0.04 | 0.79 ± 0.03 | **<0.0001** | 1.08 ± 0.04 | 0.74 ± 0.04 | **<0.0001** | 0.48 | 0.32 | 0.71 |
| Sleep | 1.13 ± 0.04 | 0.79 ± 0.03 | **<0.0001** | 1.08 ± 0.04 | 0.74 ± 0.04 | **<0.0001** | 0.48 | 0.32 | 0.71 |
| 24 h | 1.13 ± 0.04 | 0.79 ± 0.03 | **<0.0001** | 1.08 ± 0.04 | 0.74 ± 0.04 | **<0.0001** | 0.48 | 0.32 | 0.71 |
| **EE** |  |  |  |  |  |  |  |  |  |
| Day | 8.32 ± 0.15 | 8.52 ± 0.13 | **0.0142** | 8.24 ± 0.16 | 8.56 ± 0.18 | **0.0021** | 0.71 | 0.86 | 0.39 |
| Night | 5.43 ± 0.09 | 5.65 ± 0.10 | **0.0001** | 5.46 ± 0.11 | 5.66 ± 0.13 | **0.0017** | 0.82 | 0.97 | 0.99 |
| Sleep | 5.17 ± 0.09 | 5.39 ± 0.09 | **0.0010** | 5.16 ± 0.11 | 5.30 ± 0.13 | **0.0009** | 0.96 | 0.93 | 0.82 |
| 24 h | 7.14 ± 0.12 | 7.36 ± 0.12 | **0.0005** | 7.12 ± 0.14 | 7.38 ± 0.15 | **0.0008** | 0.88 | 0.90 | 0.55 |
